# Tumor immunologic heterogeneity influences response to radiation and combination immunotherapy

**DOI:** 10.1186/2051-1426-3-S2-P345

**Published:** 2015-11-04

**Authors:** Todd Aguilera, Marjan Rafat, Mihalis Kariolis, Rie von Eyben, Edward Graves, Amato Giaccia

**Affiliations:** 1 Stanford Department of Radiation Oncology, Stanford University School of Medicine, Stanford, CA, USA

## 

Clinical trials of CTLA-4 and PD-1/PD-L1 antibodies (Ab) have shown responses limited to 10-30% of patients. Increasing evidence suggests radiation (RT) can enhance responses to checkpoint therapies. However, understanding of tumor-derived factors that influence response is limited, and many preclinical models are designed to be immunogenic. We sought to develop a model of immunologic heterogeneity to study factors that influence responses to RT and immunotherapy. Two tumor clones Py117 and Py8119 derived from the PyMT mammary carcinoma in C57Bl/6 mice engraft efficiently and upon RT the Py117 but not Py8119 tumors regressed. There was no significant difference detected in radiosenstivity by clonogenic survival, hypoxia by pimonidazole staining, or angiogenesis through Meca32 staining. Tumor dissociation 10 days after 12 Gy treatment revealed a significant increase in CD45+ leukocytes, CD8+ T cells and decreased CD4+ T cells, myeloid derived suppressor cells, and macrophages in Py117 tumors but minimal changes in Py8119 tumors. Secondly, there was elevated MHCI and induced PD-L1 expression in Py117 compared to Py8119 tumors suggesting a greater response may be obtained with immunotherapy. Mice treated with 12 Gy, 12Gy + PD-1 Ab, and a single dose of CTLA-4 Ab 3 days prior to RT and PD-1 therapy revealed prolonged response with RT + PD-1 and RT + PD-1 + CTLA-4 groups, significant by repeated measures analysis. Notably 2/8 tumors treated with RT + PD-1 and 6/8 mice treated with RT + PD-1 +CTLA-4 were undetectable at day 81. Implantation of tumors with different ratios of Py117 and Py8119 cells revealed that 50% inoculation of Py117 cells leads to an immune response against both tumor clones characterized by greater tumor MHCI expression, CD8+ T cell infiltration and decreased macrophage infiltration. We hypothesized that treating the unresponsive Py8119 tumors with CD40 agonist would replicate the findings of the mixing experiment. CD40 Ab in combination with 20 Gy RT resulted in a greater antitumor response with significant decrease in tumor size not seen in either treatment alone. Evaluation of the tumor microenvironment revealed MHCI expression was enhanced, macrophage infiltration was decreased and CD8+ T cells were increased. These results support that tumor immunologic heterogeneity can influence immune responses after radiation and targeting suppressive factors in the tumor microenvironment can enhance responses. This model of heterogeneity without genetic manipulation is ideal to study tumor-derived factors that enhance or suppress the immune response after RT and could inform clinical approaches to radiation and immunotherapy combinations.

